# Primary extramammary Paget disease of the male genitalia with very late (11-year) recurrence

**DOI:** 10.1016/j.jdcr.2026.05.013

**Published:** 2026-05-11

**Authors:** Gina Niscita Amisesani, Indira Utami Yusuf, Alessandro Alfieri, Verdy, Nurwestu Rusetiyanti, Paranita Ferronika, Hanggoro Tri Rinonce, Maurice AM. van Steensel, Dyah Ayu Mira Oktarina

**Affiliations:** a Department of Dermatology and Venereology, Faculty of Medicine, Public Health and Nursing, Universitas Gadjah Mada, Yogyakarta, Indonesia; b Dr. Sardjito Hospital, Yogyakarta, Indonesia; c Department of Anatomical Pathology, Faculty of Medicine, Public Health and Nursing, Universitas Gadjah Mada, Yogyakarta, Indonesia; d Universitas Gadjah Mada Academic Hospital, Yogyakarta, Indonesia; e Lee Kong Chian School of Medicine, Nanyang Technological University (NTU), Singapore

**Keywords:** dermoscopy, extramammary Paget disease, immunohistochemistry, male gentalia, primary extramammary Paget disease

## Case description

**Fig 1:**
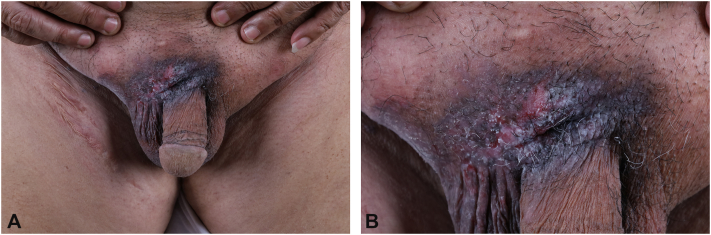
**A** and **B,** Erythematous and hyperpigmented patches and plaque, partial erosion, and moist appearance.

**Fig 2:**
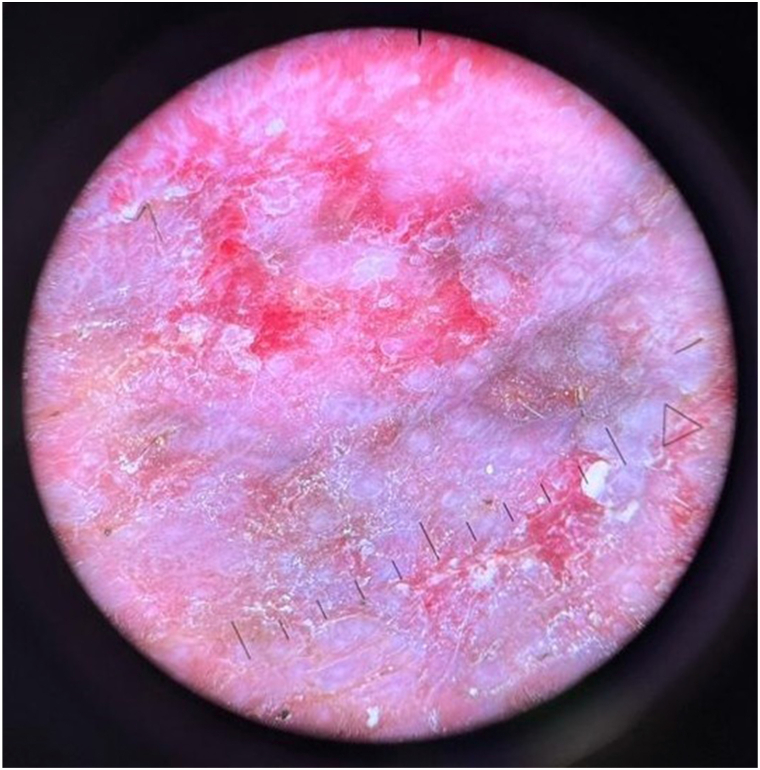
Dermoscopy shows a milky-red structureless background with white superficial scales and several shallow erosions.

A 67-year-old male presented with erythematous, pruritic patches on the pubic region. Eleven years previously, he had undergone surgical excision of a similar lesion diagnosed as extramammary Paget disease (EMPD). The lesion recurred at the same site on the right pubic region 1 year prior to presentation and thence persisted continuously without improvement, with gradual enlargement. A solitary erythematous, moist, pruritic plaque with ill-defined borders and focal erosion measuring approximately 10 × 7 cm was observed on the pubic region (Fig 1). No palpable inguinal lymphadenopathy was found. Dermoscopy revealed a milky-red structureless background with polymorphous vascular (not clearly captured in the clinical image). White superficial scales, milky-white amorphous areas, and shallow erosions were identified (Fig 2). The patient had a history of hypertension and benign prostatic hyperplasia. Blood tests, including tumor markers (Prostate-Specific Antigen/PSA, Carcinoembryonic Antigen/CEA), were within normal limits. The Multi-Slice Computed Tomography of lower abdomen showed no signs of lymphadenopathy or metastasis.

**Fig 3:**
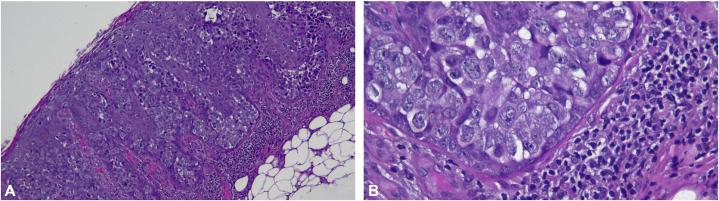
Histopathological examination of skin lesion in the pubic area shows **(A)** epidermal infiltration of atypical cells arranged in a pagetoid pattern. The upper dermis shows dilated blood vessels and diffuse inflammatory cell infiltration (Hematoxylin-Eosin, 100×). **B,** The cells are pleomorphic, with abundant clear to eosinophilic vacuolated cytoplasm and prominent nucleoli. (Hematoxylin-Eosin, 400×).

**Fig 4:**
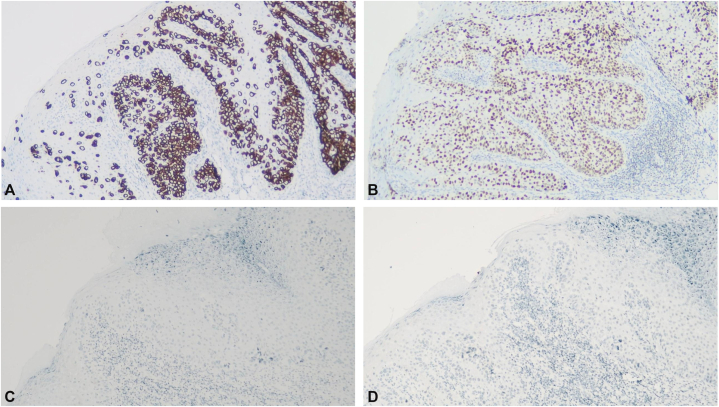
Immunohistochemical examination of skin lesion in the pubic area shows **(A)** strong membranous and cytoplasmic CK7 (+) staining in all tumor cells and **(B)** strong nuclear GATA3 (+) staining in all tumor cells, without **(C)** CK20 and **(D)** GCDFP-15 staining observed in all tumor cells.

Histopathological examination of skin biopsy on the pubic region showed parakeratosis and pagetoid intraepithelial proliferation of atypical cells with abundant pale cytoplasm and prominent nucleoli. The cells extended to the basal layer and hair follicles. The upper dermis showed dilated blood vessels and a diffuse inflammatory infiltrate predominantly composed of lymphocytes and histiocytes (Fig 3). The immunohistochemical panel demonstrated strong positive staining for CK7 and GATA3, but none for GCDFP-15 and CK20, supporting a diagnosis of primary EMPD in the appropriate clinicopathologic context (Fig 4). Melanocytic markers (eg, S100 and HMB-45) were not tested for, as findings were not suggestive of a melanocytic lesion.

**Question: Which of the following is the most appropriate management strategy to reduce recurrence in primary EMPD?**

**A.** Topical corticosteroid
**B.** Wide local excision
**C.** Mohs’ micrographic surgery
**D.** Observation only
**E.** Topical 5-fluorouracil

**Answers:**

**C.** Mohs’ micrographic surgery – Correct. Mohs’ micrographic surgery is the preferred treatment because of its precise margin control and lower recurrence rates.

## Discussion

Primary EMPD is a rare adenocarcinoma of apocrine gland–bearing skin, with an incidence of 0.1 to 2.4 cases per million person-years, most commonly involving the genital region.^1^^,^^2^ EMPD usually presents as a persistent, erythematous, and pruritic plaque.^1^ Dermoscopy reveals a milky-red, structureless background, polymorphous vessels, and white superficial scales.^1^ These features help differentiate EMPD from inflammatory dermatoses such as eczema and fungal infections. The median time to recurrence rate varies among studies, ranging from 3 to 3.5 years.^3^ The 11-year interval observed in our patient highlights the importance of long-term follow-up, even after potentially curative excision.

Histopathologically, EMPD shows intraepidermal proliferation of cells with cytonuclear atypia, abundant pale cytoplasm, and prominent nucleoli, scattered singly and in small nests throughout the epidermis, frequently extending above the basal layer. Immunohistochemistry (IHC) is essential for diagnosing EMPD and determining whether it is primary or secondary. Primary EMPD typically stains for CK7, while GCDFP-15 expression is variable.^4^ GATA3 is almost always expressed in primary EMPD.^5^ CK20 expression raises suspicion for secondary colorectal or urothelial origin.^1^ In our case, CK7 positivity, CK20 negativity, and GATA3 expression support a primary cutaneous origin.

Mohs micrographic surgery is the preferred treatment because of its precise margin control and lower recurrence rates.^1^, ^2^, ^3^ As we have shown, long-term surveillance may be warranted.^1^ Recurrent EMPD generally has a favorable prognosis in the absence of invasion or metastasis.

## Conflicts of interest

None disclosed.

## References

[1] Ishizuki S., Nakamura Y. (2021). Extramammary Paget's disease: diagnosis, pathogenesis, and treatment with focus on recent developments. Curr Oncol.

[2] Yen C.H., Lee C.H., Ho J.C. (2022). Extramammary Paget’s disease: a retrospective study in a medical center in Taiwan. Dermatol Sin.

[3] Cho A., Kim D.Y., Suh D.S. (2023). Outcomes and prognostic factors of surgically treated extramammary Paget's disease of the vulva. J Gynecol Oncol.

[4] Sainath V., Shivam R., Mallika G., Gautam T., Sarita P. (2024). Extramammary Paget's disease of the penis and scrotum. Indian J Surg Oncol.

[5] Zhao M., Zhou L., Sun L. (2017). GATA3 is a sensitive marker for primary genital extramammary Paget disease: an immunohistochemical study of 72 cases with comparison to gross cystic disease fluid protein 15. Diagn Pathol.

